# David S. Miller: Scientist, Mentor, Friend—a tribute and thank you

**DOI:** 10.1186/s12987-020-00220-5

**Published:** 2020-09-14

**Authors:** Björn Bauer, J. Larry Renfro, Karl J. Karnaky, Rosalinde Masereeuw, Gert Fricker, Ron E. Cannon, Anika M. S. Hartz

**Affiliations:** 1 Department of Pharmaceutical Sciences, College of Pharmacy, University of Kentucky, 333 Sanders-Brown Center on Aging, 800 S Limestone, Lexington, KY 40536-0230 USA; 2 Department of Physiology and Neurobiology, University of Connecticut, Storrs, CT USA; 3 Department of Regenerative Medicine and Cell Biology, Medical University of South Carolina, Charleston, SC USA; 4 Division of Pharmacology, Utrecht Institute for Pharmaceutical Sciences, Utrecht, The Netherlands; 5 Institute of Pharmacy and Molecular Biotechnology, University of Heidelberg, 69120 Heidelberg, Germany; 6 Signal Transduction Laboratory, National Institute of Environmental Health Sciences, National Institutes of Health, Research Triangle Park, NC USA; 7 Sanders-Brown Center on Aging, University of Kentucky, Lexington, KY USA; 8 Department of Pharmacology and Nutritional Sciences, University of Kentucky, Lexington, KY USA

## Abstract

David S. Miller was Acting Scientific Director of the Division of Intramural Research at the National Institute of Environmental Health Sciences, National Institutes of Health, and Head of the Intracellular Regulation Group in the Laboratory of Toxicology and Pharmacology before he retired in 2016. David received his Ph.D. in biochemistry from the University of Maine in 1973. David was a Group Leader at the Michigan Cancer Foundation before joining the NIEHS in 1985. His research covered a wide range from renal excretory transport mechanisms to regulation of transporters at the blood–CSF and blood–brain barriers, from fish, amphibians and birds to mammals. David was an outstanding scientist with irresistible enthusiasm for science and an incredible ability to think outside the box while being an exceptional mentor and friend.

## Introduction

David S. Miller’s research career maintained a central theme of understanding how the body protects itself from toxic chemicals. This line of research was initiated during his graduate studies where he characterized the transport of toxic molecules, which evolved into studies on solute transport in various model systems. In the mid-1970s, David’s work in Dr. William B. Kinter’s laboratory linked the thinning of duck eggshells to exposure to the pesticides dichloro-diphenyltrichloroethane (DDT) and dichlorodiphenyldichloroethylene (DDE) [1, 2]. To understand how the body protects and removes toxic molecules led David to first investigate transporters in fish, crabs, and sea birds. In 1985, David was recruited to the National Institute of Environmental Health Sciences (NIEHS), National Institutes of Health (NIH) as an Expert Research Physiologist in the Laboratory of Cellular and Molecular Pharmacology. Here, David and John Pritchard resumed their scientific collaboration, begun in the early 1970s at the Mount Desert Island Biological Laboratory (MDIBL), that would continue for the rest of their careers. In their studies, David and John utilized different species including teleosts, *Xenopus laevis*, crabs, flounder, rats and mice and various model systems including oocytes, renal proximal tubules, isolated membrane vesicles and isolated brain capillaries. Their collaborative research focused on organic anions and cations and led to the major discovery that anion transport was coupled to the membrane potential through dicarboxylate exchange (mostly α-ketoglutarate) afforded by the electrogenic, sodium/potassium ATPase pump [3]. Throughout the 1990s, David continued to investigate the regulation of organic anion transport in *Xenopus* oocytes and fish kidneys using killifish as a model organism. In the late 1990s, his interest shifted toward transport processes in the blood-CSF barrier (choroid plexus) and the blood–brain barrier. David recognized that confocal microscopy had immense value for studying transport processes and the confocal microscope became an integral tool in his research. By the early 2000s, researchers had identified and cloned numerous ABC transporters including David’s three favorites: P-glycoprotein (P-gp, MDR1, ABCB1), breast cancer resistance protein (BCRP, ABCG2), and multidrug resistance-associated protein 2 (MRP-2, ABCC2). David dedicated the final years of his career to identify intracellular signaling pathways responsible for the regulation of these transporters at the blood–brain barrier and contributed several seminal discoveries to the field.

## Main text

### Education, career, and scientific contribution at a glance

**Fig. 1:**
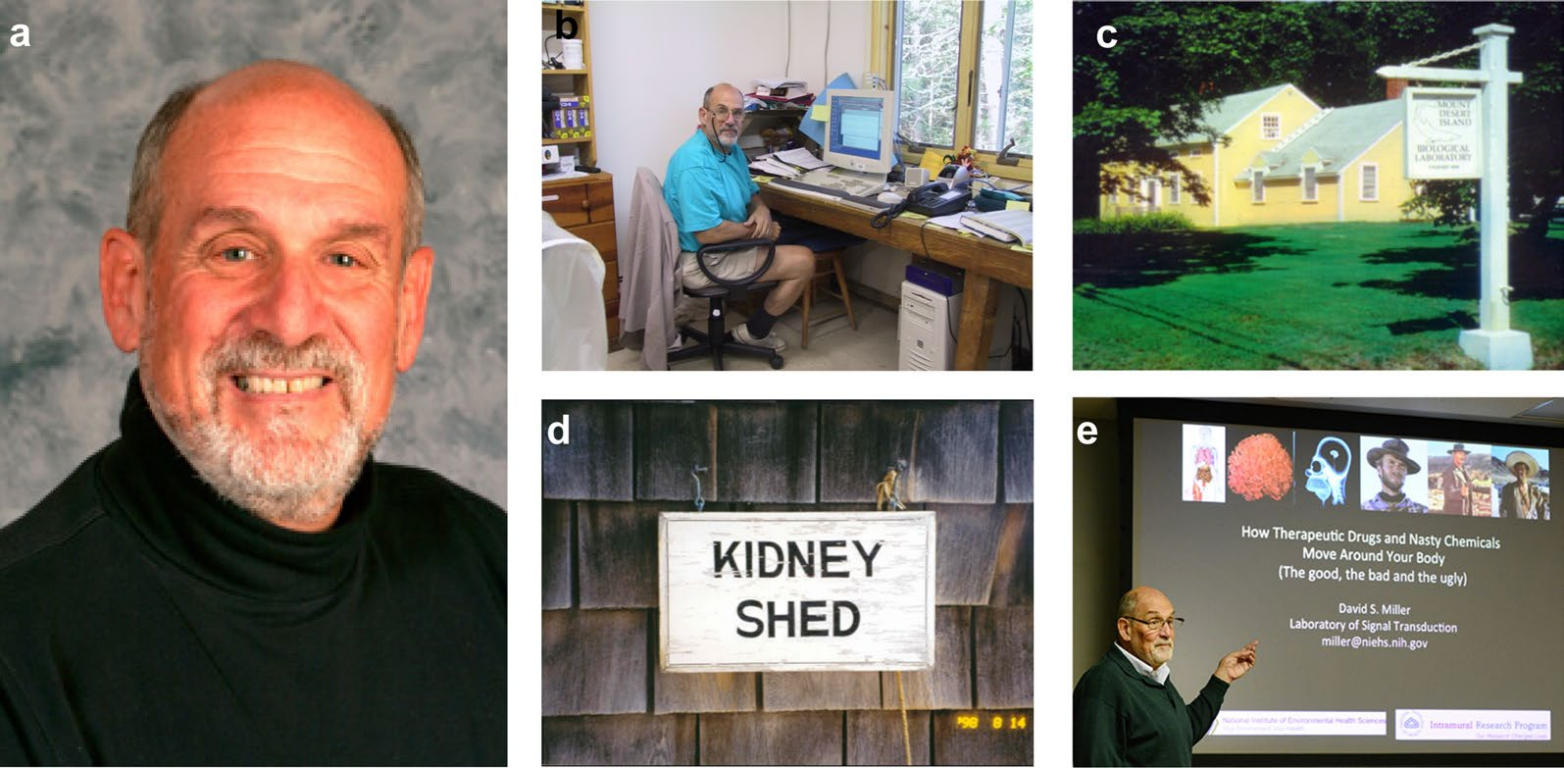
David S. Miller. **a** David S. Miller in 2010. **b** David in his laboratory at the MDIBL. **c** Mount Desert Island Biological Laboratory, MDIBL, Salsbury Cove, Maine. **d** Door sign on David’s laboratory at the MDIBL. **e** David giving a presentation entitled “*How Therapeutic Drugs and Nasty Chemicals Move Around Your Body (The good, the bad and the ugly)*” as part of the NIEHS “*Big Picture, Small Talk*” seminar series December 9, 2014 (Courtesy Steve McCaw and David Miller; https://factor.niehs.nih.gov/2015/1/inside-bigpicture/index.htm)

David Samuel Miller was born on July 24, 1945 in Brooklyn, New York City and attended Abraham Lincoln High School in Brooklyn, NY (Fig. 1a). In 1966, David received his BS in Chemistry from the Brooklyn College, Brooklyn, New York. From 1967 until 1970, David pursued graduate studies in physical organic chemistry at the City University of New York. Shortly after, David joined Dr. Joseph Lerner’s laboratory at the University of Maine, Orono, Maine, as a Ph.D. student. During his thesis work, David also attended seminars at the Mount Desert Island Biological Laboratory (MDIBL), Salsbury Cove, Maine, where he was introduced to John Pritchard, who was a MDIBL post-doctoral fellow at that time. David obtained his Ph.D. in Biochemistry from the University of Maine in 1973 and accepted a post-doctoral position at MDIBL in the laboratory of Dr. William B. Kinter. From 1976–1978, David continued at MDIBL as an Associate Research Scientist and then from 1978–1981 as a Research Scientist (Fig. 1b–d). During this time, David met Joseph Fenstermacher, who worked on the diffusion of molecules within the brain extracellular space and blood–brain barrier properties in health and disease [4]. In 1981, David joined the Michigan Cancer Foundation in Detroit, MI as an Assistant Member in the Department of Physiology and Biophysics. He became Chief of the Laboratory of Metabolic Control in 1982. In 1985, John Pritchard recruited David to the NIEHS as an Expert Research Physiologist in the Laboratory of Cellular and Molecular Pharmacology working under Martin Rodbell, a 1994 Nobel Laureate in Physiology or Medicine. At the NIEHS, David advanced his scientific career and became PI and Head of the Intracellular Regulation Group in 1988, Chief of the Laboratory of Toxicology and Pharmacology and Director of the Environmental Toxicology Program in 2010, and ultimately served as the NIEHS Acting Scientific Director of Intramural Research 2010–2011 (Fig. 1e). David remained Senior Investigator and Head of the Intracellular Regulation Group until he officially retired in 2016.

### Scientific career—from chickens to regulatory pathways at the blood–brain barrier

#### Postdoctoral work until mid-1990s

##### Early post-doctoral and research scientist years at the Mount Desert Island Biological Laboratory

Notes from Larry Renfro: David joined the Kinter laboratory at MDIBL in 1973 as a postdoc, the year before I moved to the University of Connecticut, but that overlap was sufficient to cement a career long respect for and interaction with him [5–9]. David’s biochemistry background coupled with the amino acid transport work that he and Lerner did at the University of Maine prepared him perfectly for work with the Kinter group. As a result, he had an impressively productive post-doctoral period, foretelling the superb contributions in all the later years.

With William Kinter and David Peakall, David produced several papers on eggshell thinning in duck, including two *Nature* papers and one in *Science* [1, 2, 10–12]. Toxicity of the pesticide dichloro-diphenyltrichloroethane (DDT) to humans and animals was beginning to be understood by the mid-1940s. Subsequently, effects of DDT/DDE (dichlorodiphenyldichloroethylene) were directly linked to population declines in certain avian species, especially the raptors. Stickel’s 1975 [13] assertion that DDE was a potent inhibitor of eggshell thinning remains accurate today, and the underlying cause was shown by Miller et al. [2] in their classic Nature papers on the subject in 1975–1976 [1]. Their data showed that Ca^2+^-ATPase in the oviduct positively correlated with shell thickness. Whereas the shell gland Ca^2+^-ATPase activity was not particularly higher than in liver or kidney, it was far more sensitive to DDE. They found a cause-and-effect relationship strongly suggesting that DDE inhibited duck shell gland Ca^2+^-ATPase. Species sensitivity varies widely, but this work was a hallmark for in depth physiological and endocrinological understanding of the underlying causes of this environmental tragedy.

David was involved in eight more papers focused on studying the effects of ingesting small amounts of crude oil on key physiological processes in sea birds [14–21]. At the time, it was well-known that crude oil affects the ability of sea birds to fly, and as they preen to remove it, ingest the oil coating their feathers, thus exposing the digestive tract, liver, and kidney to potential damage due to dehydration and metabolic imbalance.

David, as part of the Kinter and Peakall collaboration, used gull (*Larus argentatus*) as a model to study crude oil toxicity in sea birds. While it was known that crude oil spills caused acute toxicity and death of waterfowl, the effects of small amounts of ingested crude oil as a result of preening could now be tested revealing effects on long-term survival and potential for ultimate lethality. Again, there was a heavy emphasis on intestinal transport and Na^+^/K^+^-ATPase activity in the nasal salt gland. The work showed definitively that small dose exposure to crude oil through ingestion by sea birds produced multiple sublethal disruptions of metabolism and osmoregulatory capacity.

Since crude oil is a complex mixture of substances from potential endocrine disruptors to heavy metals [18], David transitioned to model substrates and transport models to better define the mechanisms of toxicity of specific substances. Chuck Holiday and others worked with David to develop the crab urinary bladder as a model to examine the mechanism of organic anion transport and later organic cation transport [16, 22–27]. In a series of papers, they showed an inwardly directed, sodium-dependent (indirect) organic anion pump on the serosal membrane, cellular accumulation, and a facilitated step at the luminal surface.

Another environmental toxin that drew David’s attention was mercury, the third most toxic and persistent environmental chemical hazard. The oceans are the major source of the most toxic form of mercury, methyl mercury, and David exploited several marine physiological models to start defining the mechanism of toxicity, especially related to epithelial transport [6, 28, 29]. He showed that a low concentration of mercury reduced organic anion transport in flounder renal tubule by both inhibiting Na^+^/K^+^-ATPase and increasing membrane ionic permeability.

In summary, David’s early work greatly increased our understanding of epithelial organic anion and cation transport processes and mechanisms of toxicity of environmental hazards.

##### Postdoctoral years at the Mount Desert Island Biological Laboratory

Notes from Karl Karnaky, Jr.: I first met David when he joined Dr. Kinter’s group as a Postdoctoral Research Associate at the Mount Desert Island Biological Laboratory (MDIBL) in Salsbury Cove in the summer of 1973, shortly after he had completed his Ph.D. in Biochemistry at the University of Maine in Orono. In what would be a harbinger of the publication rate he achieved during his career; David had already published five papers when he arrived at MDIBL [30–34]. I had not known David more than 2 min when he told me his middle initial, “*S”*, stood for “*Superb*”. That was my introduction to David’s humorous side, which I have enjoyed all these years. We have been sharing and still are sharing a ton of jokes up until this present day. This “superb” label turned out to be prophetic as David published a long list of important papers.

During David’s 3 years as a postdoctoral research associate, I was able to interact with him on a daily basis. Although most of David’s research interests during my postdoctoral years at MDIBL (1972–1976) were different from mine, I did get the chance to collaborate with him and Larry Renfro on a study in which we localized the critical transporting enzyme, Na^+^/K^+^-ATPase, in winter flounder urinary bladder using ^3^H-ouabain autoradiography. During the very rough draft stage of writing, one of the three authors submitted a very long paragraph that lumbered and collapsed of its own accord. David wrote next to the paragraph the phrase: “*E pluribus unum*” (“One out of many”).

The specific projects that David worked on starting as a postdoctoral fellow focused on transport processes, the cause of eggshell thinning, and the effects of various xenobiotic molecules on biological processes. David started this long list of publications with a 1975 paper in Nature [1]. This study focused on prolonged eggshell thinning caused by DDE in the duck. It was the start of a productive collaboration with David Peakall and William B. Kinter.

I left MDIBL for my first position at Temple Medical School in Philadelphia in September 1976 but came back for summer research until 1980. By that time, I was working on a separate project and did not collaborate with David. I returned to summer research in 1989 with a Markey Foundation Grant from MDIBL and was able to return for summer research there a number of years until 2002 with the help of the Salsbury Cove Research Fund. By this time, David was deeply involved in organic anion secretion processes and was utilizing the powerful tool of the confocal fluorescence microscope. This instrument could detect and help quantify transport of fluorescent xenobiotic molecules and David was a master at extracting data from biological tissues with this device. David, Rosalinde Masereeuw, John Henson, and I exploited this technique to detect the excretory transport of xenobiotics by dogfish shark rectal gland tubules [5]. We continued our studies of the shark rectal gland tubules with a paper on the regulation of a specific xenobiotic transporter, MRP2 [35]. During some of these summers, we also examined xenobiotic transporter activity in cricket Malpighian tubules. These studies culminated in a review article entitled “*The xenobiotic transporter, MRP2, in epithelia from insects, sharks, and the human breast: implications for health and disease”* [36]. We found that Mrp2 is present at the apical membrane of human breast ductules suggesting that Mrp2 transports carcinogens into the lumens of these ductules. Collaborating with David helped me develop some of my ideas and consequently secure several grants on this topic. In 2001, I invited David to give the keynote speech at an annual mini symposium, “*Epithelial metabolism and transport of xenobiotic and endogenous molecules*” that I organized in my Cell Biology and Anatomy Department at the Medical University of South Carolina.

Together, in the years from 1975 to the mid-1990s, David focused his research on excretory transport of xenobiotics in kidney, intestine, and urinary bladder of various animal models. During that time, David worked with numerous collaborators including Peakall, Kinter, Guarino, Pritchard, Holliday, Hallett, Barnes, and others including myself.

#### The 1990s to The Millennium: head of the Intracellular Regulation Group at NIEHS

##### Studying renal organic anion and cation uptake

Notes from Rosalinde Masereeuw and Gert Fricker: In the 1990s, David’s research was focused on transport processes in the kidney, and he was one of the first researchers combining functional and mechanistic aspects of transport processes in intact kidney tubules [26, 37]. Kidney tubule secretion appeared to be a two-step process mediated by carrier proteins on basolateral plasma membranes of the tubular epithelium and the luminal membranes. In addition, David showed that metabolic energy is required to pump organic cations across a cellular membrane against an electrochemical gradient—a landmark in transporter research. Using renal tubules from Southern flounder and killifish, David, John Pritchard and colleagues elucidated the relationship between organic cation uptake and basolateral membrane potential [38]. These studies were extended to organic anions in crab urinary bladder, which has a simple, flat-sheet epithelium that is structurally and functionally similar to vertebrate renal proximal tubule [27]. Subsequently, David and colleagues conducted experiments with isolated basolateral (BLM) and brush-border membrane (BBM) vesicles, both exhibiting Li^+^-sensitive, Na^+^-coupled glutarate uptake [39]. As in vertebrate renal proximal tubule, *p*-aminohippurate (PAH) was driven by exchange with (α-keto-)glutarate in bladder BLM. However, indirect coupling to Na^+^ could potentially also drive uphill PAH transport in intact epithelium. To study the role of this mechanism in net transepithelial PAH secretion, David and John Pritchard used renal proximal tubules from Southern flounder [40]. They found that tubules exhibited both Na^+^/glutarate uptake and glutarate^+^/PAH exchange. To determine if indirect coupling affected net secretion and tissue accumulation, David and John Pritchard measured the steady-state accumulation of the anionic fluorescent dye, fluorescein (FL), and demonstrated that glutarate stimulated uphill FL entry into tubular cells and also stimulated active FL secretion into the tubular lumen. Based on this observation David concluded that FL follows the route of PAH transport. It is worth noting that David used epifluorescence microscopy and video-image analysis for these studies, which became instrumental in David’s future work.

**Fig. 2:**
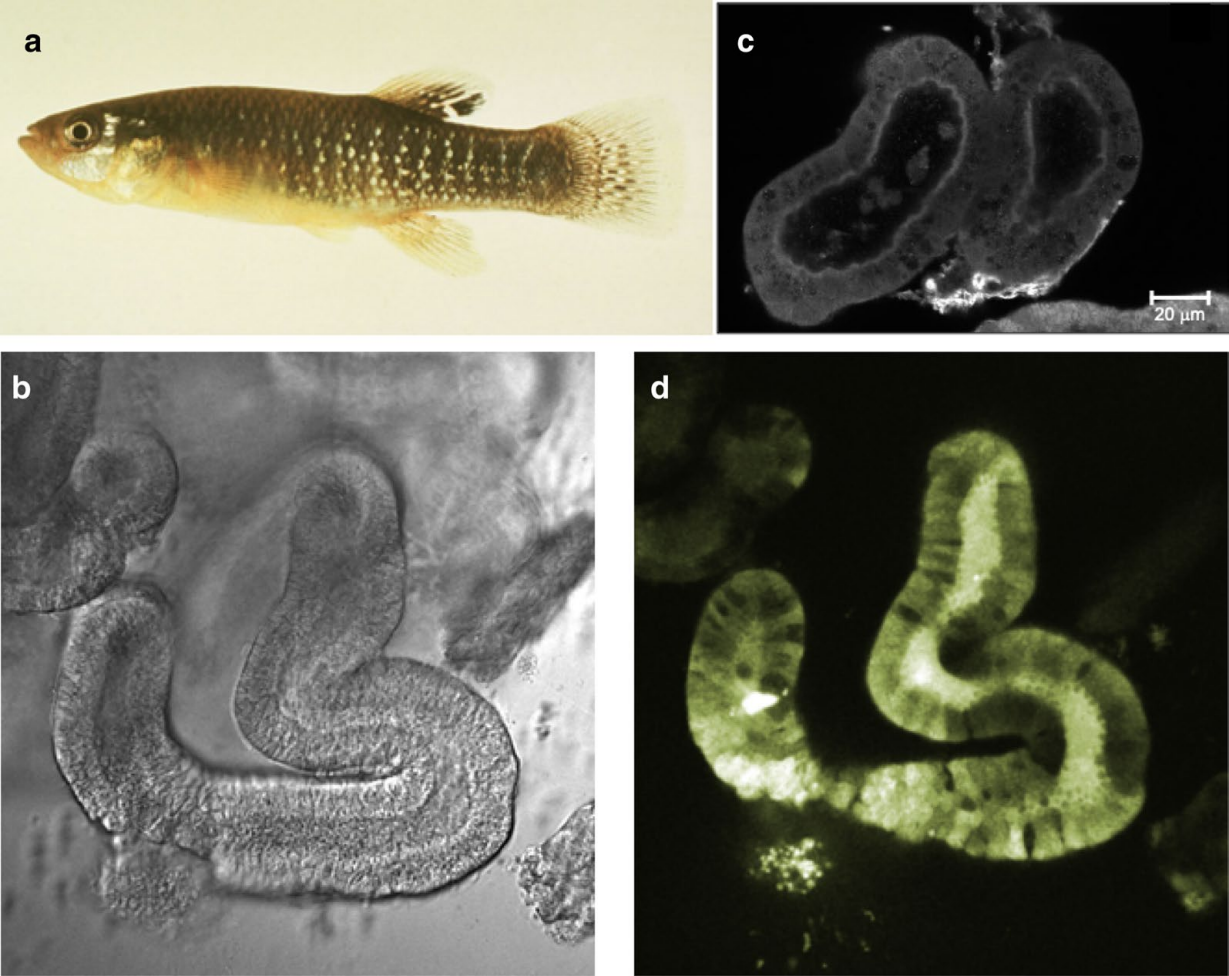
Killifish. **a** Killifish (*Fundulus heteroclitus*). **b** Transmitted light image of an intact killifish tubule. **c** Confocal image showing a killifish renal proximal tubule stained for Mrp2; [63]. From the Journal of Pharmacology and Experimental Therapeutics with permission. **d** Confocal image showing accumulation of fluorescein-methotrexate (FL-MTX) in the lumen of a killifish renal proximal tubule [58]

To investigate intracellular sequestration of organic anions, David and colleagues studied the distribution of FL in crab urinary bladder, cultured opossum kidney cells, and intact killifish (*Fundulus heteroclitus*) proximal tubules (Fig. 2; [41–43]). At that time, I, Rosalinde Masereeuw, was a Ph.D. student at Radboud Universitiy Medical Center in Nijmegen, the Netherlands and contacted David to spend time in his laboratory. My observations of FL accumulation in rat kidney proximal tubule cells contradicted his observations in other models; we solved this collaboratively [44].

##### Moving to apical transporters in the kidney

In 1994, David and I, Gert Fricker, University of Heidelberg, Germany, had a unique opportunity: we received a fluorescent derivative of the immunosuppressant cyclosporin A, NBD-cyclosporin A (NBD-CSA), that was custom-synthesized by Dr. Roland Wenger, Sandoz Pharma AG, Basel, Switzerland. The experiments that followed were the beginning of a long-lasting series of studies that continue until today. NBD-CSA turned out to be the ideal tool to visualize transport mediated by P-glycoprotein (P-gp), an ABC efflux transporter that had shortly prior been identified to transport cyclosporin A. In joint studies at the MDIBL, David, Gert Fricker and colleagues measured NBD-CSA transport in killifish proximal tubules [45] and observed that NBD-CSA accumulation in tubular lumens was time-dependent, saturable, and in steady-state, NBD-CSA fluorescence intensity in tubular lumens was 2–3 times higher than fluorescence in renal cells, which appeared to be due to P-gp.

Furthermore, we investigated the renal excretion of a fluorescent rapamycin derivative in isolated killifish kidney tubules [46, 47]. We found that excretion into the tubular lumen was blocked by metabolic inhibitors and P-gp substrates, and unlabelled rapamycin blocked P-gp-mediated secretion of NBD-CSA. We later made similar observations in killifish tubules with HIV protease inhibitors [48] and the somatostatin analogue octreotide [49, 50].

David observed this phenomenon for daunomycin in killifish renal proximal tubules [51]. At pH 8.25, daunomycin crossed the basolateral membrane of tubules by simple diffusion and was then secreted into the tubular lumen by P-gp. At pH 7.25, daunomycin was transported across the basolateral membrane by organic cation transporter-mediated uptake and simple diffusion, and was secreted into the lumen by P-gp and the organic cation/H^+^ exchanger, which was most likely what was later identified as the Multidrug and Toxin Extrusion proteins, MATE1 and MATE2-K.

With Jim Boyer, Yale Medical School, New Haven, CT, David also studied isolated hepatocytes from the marine vertebrate *Raja erinacea* (little skate; [52]). Using confocal fluorescence microscopy, David and colleagues elucidated the cellular organization of actin filaments and observed that nocodazole disrupted transcytosis of a fluorescent bile salt derivative into canalicular lumens. The polarized arrangement of microtubules, the finding of cytoplasmic dynein, and the inhibition of bile salt secretion by nocodozole were all consistent with the microtubule cytoskeleton playing a fundamental role in mediating endocytosis, transcytosis, and biliary excretion in these hepatocytes.

In skate hepatocyte clusters, David, Gert Fricker and colleagues also found that the microtubular network was involved in the secretion of the fluorescent bile salt derivative NBD-taurocholate (NBD-TC; [53]). NBD-TC uptake by skate hepatocytes appeared to be active, but not dependent on the membrane potential or Na^+^, whereas its secretion into canaliculi was driven, at least partially, by the membrane potential and was depended on intact microtubules.

Transport of organic anions remained David’s research focus. Using FL, carboxyfluorescein diacetate and bimane-S conjugates, he showed that organic anion excretion in killifish tubules is carrier-mediated and against the concentration gradient but not sensitive to the electrical potential difference across the luminal membrane [54]. By this time, we had discovered an additional transport route for organic anions that was distinct from PAH and FL. Using killifish renal proximal tubules and the larger organic anion, fluorescein methotrexate (FL-MTX, 923 Da), we observed Na^+^-independent influx and active luminal secretion that mimicked the recently identified multidrug resistance-associated protein, MRP2, another ATP-driven ABC efflux transporter (Fig. 2d). Using immunostaining, we confirmed Mrp2 expression at the luminal membrane of killifish tubules [55]. A similar transport system also appeared to be present in dogfish shark (*Squalus acanthias*) rectal gland, which is a specialized, osmoregulatory organ composed of numerous blind-ended, branched tubules emptying into a central duct [56].

In 1998, David published a key observation made in killifish tubules, which became important for our future studies on transporter regulation and signaling: Protein kinase C (PKC) regulation of organic anion transport suggesting direct or indirect regulation of organic anion transport at the basolateral membrane [5]. In addition, active excretion of daunomycin and NBD-CSA in killifish renal proximal tubules and monolayers of primary flounder kidney cells was affected by PKC activators and inhibitors indicating that P-gp-mediated xenobiotic secretion negatively correlates with PKC activity [57].

For our studies, isolated killifish proximal tubules represented a unique model to study the regulation of transporters, in particular MRP2 and P-gp. Over several summers at the MDIBL (1998–2005), we used FL-MTX and NBD-CSA to investigate the role of the vasoactive peptide endothelin-1 (ET-1) on the functional expression of the efflux pumps P-gp and Mrp2 [58–62]. The ET-1 effect on P-gp and Mrp2 was mediated by ET-1 activation of the ET(B) receptor followed by downstream signaling involving PKC, PKA, and nitric oxide (NO). Exposing renal tubules to nephrotoxicants resulted in the generation of NO and upregulation of the efflux pumps, which likely has a nephroprotective effect through mediating the excretion of harmful xenobiotics. We made this observation after short-term exposure as well as after longer-term exposures [63]. These findings suggested involvement of genomic as well as post-translational regulation of the efflux pumps in killifish kidney tubules, which was confirmed for Mrp2 several years later [64].

##### From kidney tubules to choroid plexus and brain capillaries

In 1999, David switched from liver and kidney to choroid plexus, an organ which is often referred to as the “*kidney in the brain*” due to its functional similarity to renal tissue. Together with Alice Villalobos and John Pritchard, David studied uptake and distribution of the fluorescent organic cation quinacrine in primary cultures of rat choroid plexus epithelial cells [65] and in intact choroid plexus tissue from cow [66]. Since the rat renal exchanger (rROAT1) was cloned together with Douglas Sweet and John Pritchard, [67], a rROAT1-green fluorescent protein construct was used to analyze the protein distribution directly in transiently transfected rat choroid plexus. Consistent with the functional studies, the GFP-tagged transporter was detected in apical but not basolateral membranes of the choroid plexus.

Looking back on the transporter field of the 1990s, David’s work had an enormous impact both on our understanding of mechanisms underlying renal function as well as on the development of novel tools to investigate transport phenomena in the kidney. The combination of confocal laser scanning microscopy and isolated intact kidney tubules offered completely new perspectives to investigate transport in an experimental ex vivo system that was closer to the in vivo situation as any other model used before. Thus, it was only natural to apply these approaches for other tissues, e.g., the choroid plexus and the blood–brain barrier, as well. In 2000, David and colleagues published the first paper identifying transporters and studying their function in isolated pig brain capillaries [68]. These results validated a new method of studying drug transport in isolated brain capillaries and implied that both P-gp and Mrp family members are involved in drug transport at the blood–brain barrier.

#### The 2000s until 2010

Notes from Anika Hartz and Björn Bauer: Before the year 2000, David’s work was primarily focused on renal transport, but he also had some collaborations studying the choroid plexus (the “*kidney in the brain*”). Starting in the late 1990s, David’s interest and excitement shifted toward transporters at the blood–brain barrier, which remained his main research focus until his retirement. The first studies evolved around xenobiotic transport in isolated brain microvessels from killifish using live imaging and confocal microscopy, David’s favorite and most powerful instrument in the laboratory [35, 68, 69].

**Fig. 3:**
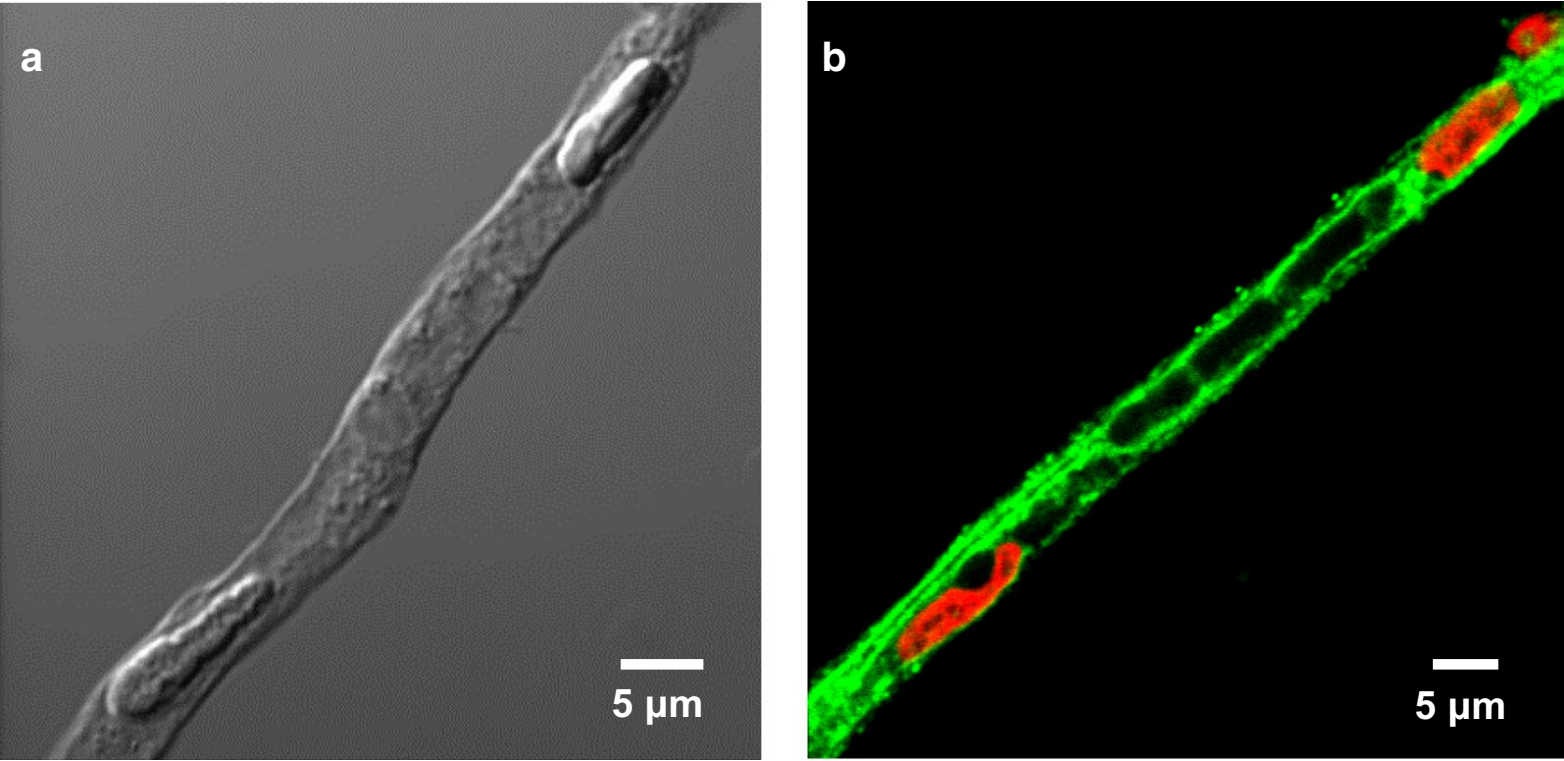
Brain capillary model. **a** Transmitted light image of an isolated rat brain capillary. **b** Confocal image of an isolated rat brain capillary immunostained for P-glycoprotein (green, nuclei counterstained with propidium iodide in red; [103]. From Pharmacological Reviews with permission

In July 2002, we—Bjoern Bauer and Anika Hartz—arrived at Raleigh-Durham International airport in North Carolina, where John Pritchard picked us up since David had already left for Maine. After settling in our new home and lab at the NIEHS, we joined David at the MDIBL for the rest of the summer working on ET-1-mediated regulation of Mrp2 in isolated killifish brain capillaries. After returning to North Carolina, our first charge in David’s laboratory was to establish a brain capillary isolation protocol for rodents. With this protocol in place, we began translating some of David’s previous work in fish and other species into isolated rat brain capillaries. This was the start of a series of studies investigating signaling pathways that regulate blood–brain barrier ABC transporters such as P-gp, BCRP and Mrp2 (Fig. 3). The first study closely followed the discoveries made by David et al. in kidney tubules showing that the hormone endothelin-1 (ET-1) regulates P-gp transport activity in isolated brain capillaries [70]. In parallel, Bjoern convinced David to look into nuclear receptor regulation of ABC transporters, an idea that was sparked by a seminar Ronald Evans, the father of nuclear receptors, held at the MDIBL that Bjoern had attended in July 2001. We found that the pregnane X receptor (PXR) is expressed at the blood–brain barrier where it regulates P-gp protein expression and transport function [71–73]. These initial studies built the framework for many following studies. Along the way we discovered that the inflammatory mediators, TNF-α and ET-1, regulate P-gp in a time-dependent and concentration-dependent manner—a finding David labeled as “*context*-*dependent regulation*” [74]. Based on these findings, David hypothesized that targeting inflammatory signaling in CNS pathologies could have a potential therapeutic benefit, which would prove correct in our later work and is still valid and part of our research to this day.

Around the same time, David started a collaboration with John Hong’s group at the NIEHS, expanding our inflammation studies to an environmental toxin—diesel exhaust. We found that diesel exhaust particles induce oxidative stress and pro-inflammatory signaling that up-regulates P-gp at the blood–brain barrier [75]. In 2005, David had a discussion with us that would prove to be critical for our careers: he pointed out that in order to be successful in securing NIH funding, we would have to start thinking about connecting our interests in blood–brain barrier transporter regulation to CNS disease. This prompted Bjoern to explore the mechanism underlying transporter regulation at the blood–brain barrier in epilepsy, an idea based on published work [76] and a seminar highlighting the role of glutamate in epilepsy. David invited Heidrun Potschka, an expert in animal seizure models, to visit the NIEHS in 2005. A first combined ex vivo*/*in vivo study followed that focused on a signaling pathway through which seizures up-regulate P-gp expression at the blood–brain barrier [77]. Based on our previous work studying the effect of inflammatory markers on transporters [70, 74], David suggested early on in the project that we also look into COX-2-mediated inflammation in epilepsy. Indeed, our data suggested that COX-2 inhibition may be one way to improve the response to antiepileptic drugs, a concept that was exciting to all of us. Several more publications around this topic followed [77–79] and this line of research eventually translated into Bjoern’s research program that is an active project in our laboratory to this day [80–82].

In 2005, one publication—Cirrito et al. [83]—raised David’s interest. In combination with the paper by Lam et al. [84] this work suggested that blood–brain barrier P-gp transports Aβ, a neurotoxin that accumulates in Alzheimer’s disease [83, 84]. David became curious and wanted to find out if we could follow P-gp-mediated Aβ transport in isolated brain capillaries. David’s initial experiments using confocal microscopy and fluorescent-labeled Aβ_40_ did not seem to work. While he lost interest, Anika decided to give the assay another try. By simply switching from Aβ_40_ to Aβ_42_, she made the assay work in isolated rat brain capillaries and brought back David’s interest. We later learned that Aβ_40_ triggers a signaling pathway that reduces P-gp transport activity explaining why David’s initial experiments “did not work” [85, 86]. This project developed into Anika’s current research program.

In a project focusing on another ABC transporter—breast cancer resistance protein, BCRP (ABCG2)—we unraveled an estrogen signaling pathway through which 17β-estradiol (E2) downregulates BCRP, another critical ABC efflux transporter at the blood–brain barrier [87, 88]. We found that E2 signals through ERbeta, PTEN/PI3K/Akt/GSK3 leading to proteasomal degradation of BCRP. Later we discovered that intervention with this pathway downregulates both BCRP and P-gp, which is a strategy we currently test in our laboratory to increase brain uptake of chemotherapeutic drugs in brain cancer.

Looking back, long days in David’s laboratory were broken up by group lunches and birthday celebrations with food and cake organized by postdocs and students. David enjoyed these social events and used them as an opportunity to connect with lab members to talk about science and more. David loved to be part of these events, but he never wanted to be the center of attention.

Weekly lab meetings were held jointly between our and John’s group. In fact, we were functioning as one big group with the luxury of having two fantastic mentors. In 2004, John Pritchard won the “*NIEHS Mentor of the Year*” award; David received the award in 2006. This award is presented by the NIEHS Trainees Assembly and honors an NIEHS scientist who has made a major impact both scientifically and personally in the training of fellows and students. This achievement highlights John and David as mentors. In the nurturing environment that John and David created, we made many fond memories. Trainees from David’s and John’s laboratories found jobs around the world and while we now live far apart from each other and are busy with our lives, we are all still connected, we talk and meet from time to time and some of us even collaborate.

During the time we were in David’s laboratory 2002–2007, David spent his summers at the MDIBL in Salsbury Cove in Maine, where we occasionally visited him to discuss data, projects and ideas. Dusty (Destiny Sykes), David’s research technician of almost 30 years, was his right and left hand in the laboratory and spent weeks of packing and shipping material and equipment to Maine each summer. Dusty was critical for David’s laboratory in many ways: she was the heart and soul of the lab and took the lead on David’s “*crazy idea experiments*” that he wanted to pursue. Dusty also helped new postdocs from all parts of the world feel welcome and at home, helped them get acquainted with daily life in the lab, and she provided critical input for the perfect Thanksgiving dinner for us foreigners.

Outside of the laboratory, David and his wife Mimi are avid horseback riders. They spent their weekends at the J&H Staples in Raleigh, North Carolina, where they boarded their horses. Both helped around the barn: they took care of the horses, cleaned the barn, enjoyed helping kids getting ready for their riding lessons, and watched and socialized with their parents during lessons. David and Mimi also took their horses out for long rides around the barn and to weekend-long riding through to nearby Virginia. They even convinced us to take up riding, which created a nice balance to the work in the laboratory. We left the NIEHS in July 2007 to take on faculty positions but kept collaborating with David on various projects while establishing our own laboratories. David hired highly productive postdocs who made significant scientific contributions in linking changes in blood–brain barrier and choroid-plexus transport to specific pathologies and xenobiotic exposures [89–96].

#### 2010 until 2015

Notes from Ron Cannon: One late afternoon while working on the confocal microscope, I—Staff Scientist in the laboratory of Raymond Tennant at the NIEHS—happened to meet David while I was trying to localize a newly discovered transporter, ABCA13, using immunohistochemistry. David was working on the confocal and was immediately interested in my findings. We talked for about an hour as he assisted me with the appropriate confocal settings to optimize my results. Within 6 months Raymond Tennant retired and David approached me with a request to join his laboratory. I gladly accepted and I worked with David until his departure in 2015. The work with David was most enjoyable and productive. David was driven by his passion for science. Numerous nights we discussed science by phone and continued the following morning. It was never a question of finding a project. The office and late-night discussions filled the project black board in his office. There was never a question of “*do we have a scientific project to study?*” It was only a question of “*which one*”.

**Fig. 4:**
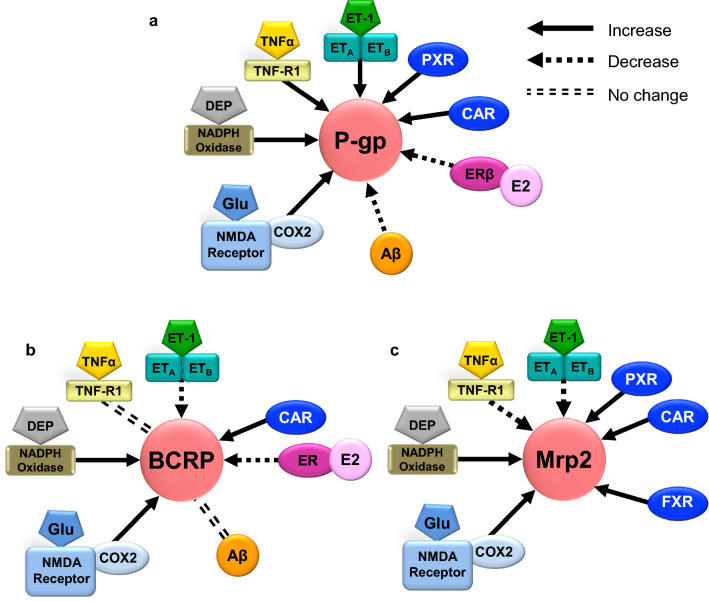
Summary of signaling pathways. Signaling pathways that regulate **a** P-gp, **b** BCRP, and **c** Mrp2 at the blood–brain barrier. Clockwise: ET-1 signaling upregulates P-gp, but downregulates BCRP and Mrp2. The nuclear receptor pregane X receptor (PXR) upregulates P-gp and Mrp2, the farnesoid X receptor (FXR) upregulates Mrp2, and the constitutive androstane receptor (CAR) upregulates P-gp, BCRP, and Mrp2. Estradiol (E2) signals through the estrogen receptor β (ERβ) to stimulate proteasomal degradation of P-gp and BCRP. Amyloid-β (Aβ) triggers degradation of P-gp but has no effect on BCRP. Glutamate released during seizures signals through the *N*-methyl-d-aspartate receptor (NMDAR) and cyclooxygenase-2 (COX-2) upregulation of P-gp, BCRP, and Mrp2. Diesel exhaust particles (DEP) activate NADPH oxidase resulting in P-gp, BCRP, and Mrp2 induction. TNF-α signaling through TNF-R1 upregulates P-gp and downregulates Mrp2, but has no effect on BCRP (Courtesy David Miller)

Most of David’s previous transporter work was focused on the blood–brain barrier transporter P-glycoprotein and how xenobiotic molecules modulated its activity. We decided to study how endogenous molecules might regulate ABC transporters like P-glycoprotein since that could be a better and more natural way in terms of treatment. We focused on how sphingolipids and ceramides regulate P-glycoprotein [97, 98]. Work in the lab progressed and we unraveled how sphingosine-1 phosphate and ceramide-1 phosphate regulate P-glycoprotein [97]. We later found that the S1P regulatory pathway was regulated upstream by a previously described TNF-alpha pathway [99]. This work led to the finding that ceramide-1 phosphate induces P-glycoprotein transport activity. Moreover, this work helped conceptualize that ABC transporters may be dynamically controlled at the activity level without the need for changes in expression [100–102]. Figure 4 highlights David’s work on the regulation of ABC transporters at the blood–brain barrier.

### David’s departure and retirement from the NIEHS

David suffered a stroke in July 2015 and officially retired in early 2016 after more than 30 years of research at the NIEHS. He was well-known and liked by everyone. He loved scientific research; it was his passion. He mentored numerous students, post bac and post docs inside and outside of his lab. All of whom still call or sometime visit the laboratory. As I came to know him personally, I discovered his love for boating and horses. He would fly in lobsters from Maine to North Carolina for the yearly Lobster Fest at the barn where he boarded his horses. Then he would cook them in large pots as everyone anxiously awaited. He was kind and fair to everyone, no matter what their “walk” in life. Now as I walk the halls of NIEHS to attend meetings or lunch in the cafeteria, two questions and one statement are made by those who approach: “*Have you talked to Dave lately? How is he doing? Tell him I asked about him and tell him we really miss him*”. I think this says it all.

## Beyond the science

Outstanding science can be summarized in a review article. We want this tribute to David to be more than a summary of professional accomplishments. The following personal memories, thoughts and thank you notes highlight what makes David special to us as a scientist, mentor, and friend.

Larry Renfro: “*Science aside, perhaps David’s most important contribution is his continuing good*-*natured irascibility, matter*-*of*-*fact no*-*nonsense approach to science, and contagious enthusiasm. Thank you, David, for all your help.”*

Karl Karnaky: *“Of all the people one meets in life only a few are really special. For me, David is one of these people* – *not only for the rewarding scientific interactions but also on a human interaction level. There were two enormous emotional situations, one for David and one for me, in which we helped each other immensely. Fortunately, our interactions continue as David and Mimi moved to Ellsworth in 2016. For about nine years now, I have been coming to Bar Harbor, about 25* *min away, and we are able to have breakfast once a week while I am here. I related several humorous stories in the text above, but I am so glad to be able to see David for a good portion of the year now. After knowing David for 45* *years I still see why he is “superb”.*

Rosalinde Masereeuw: *“I arrived in David’s lab in February 1995 as a Ph.D. student from the Netherlands who was awarded a fellowship for an international training. I could not have made a better choice in selecting a lab. David was the best mentor ever: he would patiently sit down with me to draw a serious research plan, in his own way with lots of jokes in between. His curiosity and enthusiasm was a driver in our collaborative work. He also showed how to balance between a work life and a private life. For me, he was the example of how to be a scientist and a mentor myself. Thank you, David!”*

Gert Fricker: *“I first met David 30* *years ago at the MDIBL and for me it was one of the most important encounters in my life. His outstanding ability to explain scientific coherencies together with his most helpful and patient personal attitude made him an ideal mentor and very good friend for me. David, thank you for all.”*

Anika Hartz: *“David infected me with his contagious enthusiasm for science. David’s assurance, continuous mentoring and his love and passion for science convinced me that science is the best. David introduced to me the concept of “thinking outside the box” by showing me the box I was trapped in* – *a game changer for me and most other trainees that listened carefully what he said. To complex questions David had a simple answer: “Do the right thing”* – *this could mean to repeat a time*-*consuming experiment, rewrite a manuscript or simply help a newcomer in the lab. What it gave me as a trainee was guidance, professionalism and a simple rule applicable to science and life in general. David: Thank you for doing the right thing!”*

Björn Bauer: *“My time in David’s lab was invaluable. David had the patience to listen and always gave honest feedback: he told me the things I needed to hear, not the things I wanted to hear. To me, one of the most memorable things David said was when he received the NIEHS Mentor of the Year Award. David said: “I can’t believe they let me play in the sandbox and even pay me for this!”. Thank you, David, for the unforgettable experience. Your advice resonates with me to this day!”*

Ron Cannon: “*As I reflect on my years of working with David, I recognize his greatest ability was to lead with passion. His excitement for every project in the lab was infectious. His wide smile as new and interesting data arrived was genuine and motivating. And rather than sit alone and ponder new data, David preferred open engagement and discussions. He urged us to thinking deeply about our work and to consider its meaning and implications. What a great life lesson! Thank you, David, for being a good friend, a great colleague, and teaching us how to enjoy science to its fullest!”*

John Pritchard: *“David and I first met almost 50* *years ago, when he was still a graduate student with Lerner in Orono and I was a post doc in Bill Kinter’s laboratory at MDIBL. We have worked together on and off ever since. We both gained a solid grounding in renal physiology, the comparative method, and a mechanistic approach to environmental issues with Bill. I subsequently focused on isolated membrane vesicles and molecular tools, while David developed confocal and fluorescence microscopy approaches. Together we collaborated on studies of renal function and mechanisms of environmental toxicity over many of those 50* *years. Not only did our scientific tools complement each other, but our work styles were similarly complementary. I was the more systematic, whereas David was more free ranging, ensuring that we looked at all the possibilities. It was a good run, and a fun one as well. I count myself as extremely fortunate to have had such a good friend and colleague.*

*Finally, I note that our lives have continued to parallel each other in recent years. David had his stroke and I, my cervical spinal injury. Happily, we have both made considerable progress in establishing a new normal, one that we continue to refine.”*

### David S. Miller Young Scientist Award—honoring David’s mentorship for years to come

The “*David S. Miller Young Scientist Award*” was initiated in 2016 to honor David as a mentor, as a scientist, and as a friend. David had a positive impact on the career and life of numerous students and postdocs. He encouraged young scientists to think outside the box, sparked their interest in research, gave them valuable insights into the world of science, and guided each student along the way. The “*David S. Miller Young Scientist Award*” honors David’s mentorship by supporting young scientists with promising research in the brain barriers research field.

## Data Availability

Not applicable.
